# Interruption and Non-Adherence to Long-Term Adjuvant Hormone Therapy Is Associated with Adverse Survival Outcome of Breast Cancer Women - An Asian Population-Based Study

**DOI:** 10.1371/journal.pone.0087027

**Published:** 2014-02-21

**Authors:** Kun-Pin Hsieh, Li-Chia Chen, Kwok-Leung Cheung, Chao-Sung Chang, Yi-Hsin Yang

**Affiliations:** 1 School of Pharmacy, College of Pharmacy, Kaohsiung Medical University, Kaohsiung, Taiwan; 2 Department of Pharmacy, Kaohsiung Medical University Hospital, Kaohsiung, Taiwan; 3 Division for Social Research in Medicines and Health, School of Pharmacy, University of Nottingham, Nottingham, United Kingdom; 4 Division of Breast Surgery, School of Graduate Entry Medicine & Health, University of Nottingham, Derby, United Kingdom; 5 Department of Healthcare Administration and Medical Informatics, College of Health Sciences, Kaohsiung Medical University, Kaohsiung, Taiwan; 6 Cancer Center, Kaohsiung Medical University Hospital, Kaohsiung, Taiwan; Sudbury Regional Hospital, Canada

## Abstract

This study aimed to evaluate the survival rate of women with breast cancer (BC) comparing persistence versus interruption and adherence versus non-adherence to adjuvant hormonal therapy (HT) in Asian population. Newly-diagnosed BC women from 2003 to 2010 were retrospectively identified from the Taiwan National Health Insurance Research Database. HT prescriptions were extracted to define treatment interruption and medication possession ratio. Their impacts on mortality were estimated by Cox regression with time dependent covariates. Interruption (HR: 1.32; 95% CI: 1.20, 1.46; *P*<0.0001) and non-adherence (HR: 1.45; 95% CI: 1.32, 1.59; *P*<0.0001) to adjuvant HT were significantly associated with increased mortality. Interruption to tamoxifen in younger patients and in patients receiving surgery (OP) with adjuvant chemotherapy (CT) was associated with increasing mortality rate when compared with their counterparts. Non-adherence to AIs in both younger and senior age groups and in OP with CT group also resulted in increasing risk. Treatment interruption and non-adherence to adjuvant HT were found to be associated with the increasing all-cause mortality of the Asian BC women; a greater impact of interruption and non-adherence on mortality was especially found in the younger BC population.

## Introduction

Breast cancer (BC) is the most common cancer and leading cause of cancer-related deaths for women worldwide [1]. Although a 5-year course of adjuvant hormone therapy (HT), including selective oestrogen receptor modulator (e.g. tamoxifen) and third-generation aromatase inhibitors (AIs; e.g. anastrozole, letrozole or exemestane), has been proved effective in reducing recurrence and mortality in hormone-sensitive BC [2], [3], emerging evidence has revealed a suboptimal adherence (correct dosage at the prescribed frequency) and persistence (continued therapy) to adjuvant HT [4]–[6]. Recent retrospective cohort studies conducted in Scotland [7] and the U.S. [8] also suggested that poor adherence and early discontinuation of adjuvant HT were associated with an increased risk of poor BC treatment outcomes.

Non-adherence and non-persistence to HT is a complex and multifaceted issue, and currently there is no gold standard of measures; even the conventional computation and cut-off threshold of medication possession ratio (MPR) and definition of gap period (60, 90, 180 days) varied between studies [8], [9], and thus jeopardizes the validity of any association identified between non-adherence and non-persistence with BC survival.

Current evidence on the impacts of non-adherence and non-persistence to HT on BC mortality has predominately emerged from Western countries [7], [8], where the peak age of BC diagnosis (60 to 70 years) is about 10 years later than those from Asian countries (40 to 50 years) [10]. The influence of non-adherence and non-persistence to HT on Asian BC patients has not been extensively evaluated.

In Taiwan, BC is the most common cancer and the fourth leading cause of cancer death for women. The incidence of BC increased from 40 to 56.1 per 100,000 women from 1999 to 2008, and the median age of diagnosis was 51 years [11]. BC treatment is delivered under the coverage of the Taiwan National Health Insurance (NHI), and both tamoxifen and AIs are available adjuvant HT for BC in postmenopausal women. According to the NHI reimbursement policy, tamoxifen is normally the HT agent of choice and AIs are reimbursed under a set of criteria.

The Taiwan Health Insurance Research Database (NHIRD) contains all medical claims from 1995; this longitudinal, population-based dataset serves as an appropriate resource for studying long-term HT utilization and BC treatment outcomes. Therefore, this study aimed to evaluate the impact of interruption and non-adherence to long-term adjuvant HT on survival outcome of BC women in Taiwan.

## Materials and Methods

### Study Design and Data Source

This retrospective cohort study was conducted from 2003 to 2011 using the Taiwan NHIRD after being granted ethics approval from the Institutional Review Board (IRB) of Kaohsiung Medical University Hospital (KMUH-IRB-20120047). Informed consent was waived by the IRB. Taiwan NHI is a government-run, single-payer health insurance scheme established in 1995. It covered 97.1% of the entire population of 21.86 million in 2000 and 99.6% of the entire population (23.74 million) in 2010, and provides comprehensive benefit packages for a wide range of services [12]. This study used outpatient and inpatient medical claims, and dispensing claims from community pharmacies of the NHIRD.

### Cohort Selection

The study cohort included adult (age ≥18 years old) women with newly-diagnosed BC from 2003 to 2010, without other concomitant cancers, who underwent surgical operations (breast-conserving surgery or mastectomy) as initial treatment and received at least 12-month adjuvant HT.

BC patients were identified by screening BC-related International Classification of Diseases Revision 9 codes (ICD-9 codes 174) from both the individuals’ claim data and the Registry for Catastrophic Illness (a patient file in NHIRD). The disease index date was defined as the first positive BC diagnosis date. Since the ICD-9CM code 174 may be recorded for the purpose of BC screening, therefore, a second BC diagnosis recorded within 30 days would likely indicate a positive BC diagnosis, and in this case the first diagnosis date was defined as the index date instead. In addition, patients who had ICD-9 codes for other cancers (140–208, except 174) or benign lesions (210–239) prior to the index date, and those who did not undergo surgery were excluded from this study.

From all HT (including tamoxifen, anastrozole, letrozole, and exemestane) prescribed to the study cohort, the ‘days of supply’ (dispensed days) for each prescription and the ‘prescription duration’ (duration between the first and the last prescriptions plus the last prescription’s days of supply) for each patient were calculated. Patients whose prescription duration shorter than 12 months were excluded from the study sample since their prognosis may not directly be related to the interruption and non-adherence of medication. A sub-group analysis among patients whose duration of HT was less than 12 months (data not shown in tables) indicated that these excluded patients had less follow-up time (3.5±2.3 years), higher proportion of receiving target therapy (10.9%), and received AI only (19.4%) as compared to the study sample (Table 1) of the manuscript. Therefore, these patients could be in a more serious condition at diagnosis, and might not response to their primary cancer treatments. All included patients were followed from the disease index date (BC diagnosed date) to death or the end of study for the following measures.

**Table 1:** Patients’ characteristics between OP and CT and OP without CT.

Characteristic	Total	Initial treatment		*P* value
		OP and CT	OP without CT	
Number of patients (%)	30573	21210 (69.4)	9363 (30.6)	
Follow-up time (year)				
Total (patient-years)	144438.8	101442.3	42996.5	
Mean±SD	4.7±2.1	4.8±2.0	4.6±2.2	<0.0001
Median (Q1, Q3)^a^	4.5 (2.9, 6.4)	4.6 (3.0, 6.4)	4.4 (2.7, 6.5)	
Age of diagnosis				
Mean±SD	52.1±11.6	49.8±9.7	57.4±13.5	<0.0001
Median (Q1, Q3)^a^	50.0 (44.0, 59.0)	49.0 (43.0, 56.0)	56.0 (47.0, 68.0)	
Age ranks (%)				
<50 years old	14383 (47.0)	11213 (52.9)	3170 (33.9)	<0.0001
50–64 years old	11391 (37.3)	8209 (38.7)	3182 (34.0)	
65–69 years old	2052 (6.7)	1139 (5.4)	913 (9.8)	
≥70 years old	2747 (9.0)	649 (3.1)	2098 (22.4)	
Insurance income ranks (%)^b^				
No income	9068 (29.7)	5776 (27.2)	3292 (35.2)	<0.0001
≦20000 NTD^c^	5940 (19.4)	4069 (19.2)	1871 (20.0)	
>20000 NTD^c^	15565 (50.9)	11365 (53.6)	4200 (44.9)	
CCI score (%)^f^				
0	21458 (70.2)	15717 (74.1)	5741 (61.3)	<0.0001
1	6009 (19.7)	3860 (18.2)	2149 (23.0)	
2	3106 (10.2)	1633 (7.7)	1473 (15.7)	
Patients received mastectomy (%)	21259 (69.5)	15387 (72.6)	5872 (62.7)	<0.0001
Patients received other adjuvant therapy (%)				
HT utilization pattern				
Tamoxifen only	20161 (65.9)	13057 (61.6)	7104 (75.9)	<0.0001
Tamoxifen to AIs	5401 (17.7)	4371 (20.6)	1030 (11.0)	
AIs only	3278 (10.7)	2542 (12.0)	736 (7.9)	
AIs to tamoxifen	267 (0.9)	157 (0.7)	110 (1.2)	
Multiple switches	1466 (4.8)	1083 (5.1)	383 (4.1)	
Radiation therapy	14672 (48.0)	11238 (53.0)	3434 (36.7)	<0.0001
Target therapy	1490 (4.9)	1354 (6.4)	136 (1.5)	<0.0001
HT prescription duration (year)				
Mean±SD	3.6±1.6	3.6±1.6	3.5±1.6	0.2675
Median (Q1, Q3)^a^	3.5 (2.1, 4.9)	3.5 (2.1, 4.9)	3.5 (2.1, 4.9)	
Patients whose HT started within 1 year of breast cancer diagnosed (%)	29291 (95.8)	20180 (95.1)	9111 (97.3)	<0.0001

Abbreviations: OP = operation; CT = chemotherapy; SD = standard deviation; NTD = New Taiwan Dollar; CCI = Charlson Comorbidity Index; HT = hormonal therapy; AIs = aromatase inhibitors.

a Q1: the 25th percentile, Q3: the 75th percentile.

b The income-related insurance payment category set by the Bureau of National Health Insurance in Taiwan.

c 1 NTD = 0.03 USD in 2012.

### Interruption and Non-adherence

From individual patient’s HT prescriptions issued during the study period, MPR to HT was derived from dividing patient’s ‘total days of supply’ (sum of days of supply from HT prescriptions) by ‘prescription duration’. A conventional cut-off point of MPR less than 80% was used to define ‘non-adherence’ [13].

Any gap period between two consecutive HT prescriptions for more than 180 days was defined as ‘interruption’ (i.e. non-persistence) [6]. Since Taiwan NHI reimburses maximally three-monthly refills to any prescription for chronic and stable conditions, a gap of 180 days without HT indicates that at least two clinical visits have been missed.

Patients’ adjuvant HT utilization patterns for the two HT types (tamoxifen or AIs) were also categorized into five groups, including tamoxifen only, tamoxifen switched to AIs, AIs only, AIs switched to tamoxifen, and multiple switches between tamoxifen and AIs. Switching of HT was defined as when patient received an alternative type for more than three refills (at least 84–90 days).

### Mortality Outcome

The mortality and date of death were identified from the Registry for Catastrophic Illness. Follow-up time was calculated from the disease index date to the date of death or to the end of study (31 December 2011) for censored patients.

### Analysis Variables

Adjusted covariates included age of diagnosis, income groups, Charlson Comorbidity Index (CCI) score, initial treatment strategies, HT initiated year, HT prescription duration. Patients’ age was categorized in four ranks (i.e. <50, 50∼64, 65∼69 and ≥70 years old). The three most recently updated NHI insured income ranks were used as a synonym for individual’s monthly income status. Individual’s concomitant conditions recorded within 12 months prior to index date were identified by screening the ICD-9 codes related to CCI, and then converted into a CCI score [14], and further categorized into three groups (i.e. CCI 0, 1 and ≥2).

Individual’s BC treatment strategies, including primary surgery (OP), adjuvant chemotherapy (CT), radiation therapy (RT), HT and targeted therapy (TT) were identified by corresponding medical-order and drug codes; and those recorded within 12 months posterior to index date were defined as the initial treatment strategies. Patients were stratified by whether they received adjuvant CT into two groups, i.e. OP with or without CT. Trastuzumab was the only TT reimbursed by NHI from April 2002 for HER2 positive BC in accordance with a set of stringent criteria, and thus TT was not included as an adjusting covariate due to limited prescription data.

### Statistical Analysis

The survival rate and all-cause mortality rate were compared between persistence and interruption, and between adherence and non-adherence groups. Overall survival rate was evaluated using Kaplan-Meier survival analysis and stratified by whether patients received initial adjuvant CT. The association between adjusted covariates (and the corresponding time-dependent covariates) and all-cause mortality were evaluated by both univariate and multivariable analyses using the Cox proportional hazards model, and the results were presented in hazard ratios (HR) and 95% confidence intervals (CIs).

The interruption- and non-adherence-associated HRs were also evaluated according to age (<50 or ≥50 years) and initial treatment (OP with or without CT) subgroups, and stratified in the four HT utilization patterns. Sensitivity analysis was conducted to assess the hazard ratios using various number of gaps or time to first gap (<1, ≥1, ≥2, ≥3, and ≥4 years) to define interruption, and various MPR cut off levels (50%, 60%, 70%, 80% and 90%) to define non-adherence. All analyses were conducted by using SAS version 9.2 (SAS Institutes, Inc., Cary, NC, US).

## Results

### Characteristics of Study Cohort

During the 8-year inclusion period, 58,601 adult women with newly-diagnosed BC were identified, accounting for 99.4% BC cases reported to the Taiwan Cancer Registry in the same period. Therefore, we identified the representative newly-diagnosed BC women in this study. Overall, 30,573 patients fulfilling the study criteria were included; with a mean diagnosed age of 52.1±11.6 years, of whom 47% were diagnosed at the age of less than 50 years, and the majority (70%) of the patients had no apparent co-morbidity (CCI scored 0) (Table 1).

The total follow-up time was 144,438.8 patient-years (mean: 4.7±2.1 years per patient), and the mean HT prescription duration was 3.6±1.6 years. Of the five HT utilization patterns, most patients (65.9%) received tamoxifen only. Comparing the two initial treatment groups, patients who received adjuvant CT were significantly younger (mean age: 49.8±9.7 vs. 57.4±13.5 years; *P*<0.0001), with higher proportions of patients without any comorbidity (CCI score = 0; 74.1% vs. 61.3%).

### Characteristics of Interruption and Non-adherence Groups

Patients in the interruption group (*N* = 4,565; 14.9%) were younger than those in the persistence (*N* = 26,008; 85.1%) group (mean age: 50.6±11.3 vs. 52.4±11.6 years). The interruption group also had higher proportions of patients undergoing mastectomy (73.8% vs. 68.8%), receiving CT (74.7% vs. 68.4%) and TT (13.2% vs. 3.4%); and a lower proportion receiving tamoxifen only (49.2% vs. 68.9%), as opposed to other HT utilization patterns (p<0.0001) (Table 2).

**Table 2:** Patients’ characteristics of persistence vs. interruption and adherence vs. non-adherence to hormone therapy.

Characteristic	Persistence	Interruption	*P* value	Adherence	Non-adherence	*P* value
Number of patients (%)	26008 (85.1)	4565 (14.9)		23631 (77.3)	6942 (22.7)	
Follow-up time (year)						
Total (patient-years)	118964.3	25474.4		109319.4	35119.3	
Mean±SD	4.6±2.1	5.6±1.9	<0.0001	4.6±2.1	5.1±2.1	<0.0001
Median (Q1, Q3)^a^	4.3 (2.8, 6.3)	5.6 (4.0, 7.2)		4.4 (2.8, 6.3)	5.0 (3.4, 6.8)	
Age of diagnosis						
Mean±SD	52.4±11.6	50.6±11.3	<0.0001	52.7±11.6	50.0±11.4	<0.0001
Median (Q1, Q3)^a^	50.0 (44, 59)	49.0 (43, 57)		52.7 (45, 60)	48.0 (42, 56)	
Age ranks (%)						
<50 years old	12045 (46.3)	2338 (51.2)	<0.0001	10602 (44.9)	3781 (54.5)	<0.0001
50–64 years old	9731 (37.4)	1660 (36.4)		9070 (38.4)	2321 (33.4)	
65–69 years old	1777 (6.8)	275 (6.0)		1660 (7.0)	392 (5.6)	
≥70 years old	2455 (9.4)	292 (6.4)		2299 (9.7)	448 (6.5)	
Insurance income ranks (%)^b^						
No income	7668 (54.8)	1400 (30.7)	0.0003	7115 (30.1)	1953 (28.1)	<0.0001
< = 20000 NTD^c^	4980 (35.6)	960 (21.0)		4413 (18.7)	1527 (22.0)	
>20000 NTD^c^	1336 (9.6)	2205 (48.3)		12103 (51.2)	3462 (49.9)	
CCI score (%)						
0	18198 (70.0)	3260 (71.4)	0.1229	16384 (69.3)	5074 (73.1)	<0.0001
1	5158 (19.8)	851 (18.6)		4780 (20.2)	1229 (17.7)	
2	2652 (10.2)	454 (9.9)		2467 (10.4)	639 (9.2)	
Patient had OP and CT (%)	17798 (68.4)	3412 (74.7)	<0.0001	16205 (68.6)	5005 (72.1)	<0.0001
Patient had OP without CT (%)	8210 (31.6)	1153 (25.3)		7426 (31.4)	1937 (27.9)	
Patient had mastectomy (%)	17890(68.8)	3369 (73.8)	<0.0001	16400 (69.4)	4859 (70.0)	<0.0001
Patient had other adjuvant therapy (%)						
HT utilization pattern						
Tamoxifen only	17914 (68.9)	2247 (49.2)	<0.0001	15345 (64.9)	4816 (69.4)	<0.0001
Tamoxifen to AIs	4135 (15.9)	1266 (27.7)		4337 (18.4)	1064 (15.3)	
AIs only	2697 (10.4)	581 (12.7)		2600 (11.0)	678 (9.8)	
AIs to tamoxifen	221 (0.8)	46 (1.0)		218 (0.9)	49 (0.7)	
Multiple switches	1041 (4.0)	425 (9.3)		1131 (4.8)	335 (4.8)	
Radiation therapy	12437 (47.8)	2235 (49.0)	0.1552	11311 (47.9)	3361 (48.4)	0.4197
Target therapy	887 (3.4)	603 (13.2)	<0.0001	849 (3.6)	641 (9.2)	<0.0001
HT prescription duration(year)						
Mean±SD	3.5±1.6	3.9±1.7	<0.0001	3.6±1.6	3.5±1.6	<0.0001
Median (Q1, Q3)^a^	3.4 (2.1, 4.9)	3.9 (2.5, 5.1)		3.5 (2.1, 4.9)	3.4 (2.1, 4.7)	
HT started within 1 year of breast cancer diagnosed	24982 (96.1)	4309 (94.4)	<0.0001	22735 (96.2)	6556 (94.4)	<0.0001

Abbreviations: OP = operation; CT = chemotherapy; SD = standard deviation; NTD = New Taiwan Dollar; CCI = Charlson Comorbidity Index; HT = hormonal therapy; AIs = aromatase inhibitors.

a Q1: the 25th percentile, Q3: the 75th percentile.

b The income-related insurance payment category set by the Bureau of National Health Insurance in Taiwan.

c 1 NTD = 0.03 USD in 2012.

Similar patterns were found while comparing the HT non-adherence (n = 6,942; 22.7) against the adherence (n = 23,631; 77.3%) groups. The non-adherence group had a significantly higher proportion receiving tamoxifen only (69.4% vs. 64.9%), as opposed to having other HT utilization patterns (*P*<0.0001) (Table 2).

### Survival Associated with Interruption and Non-adherence

**Figure 1:**
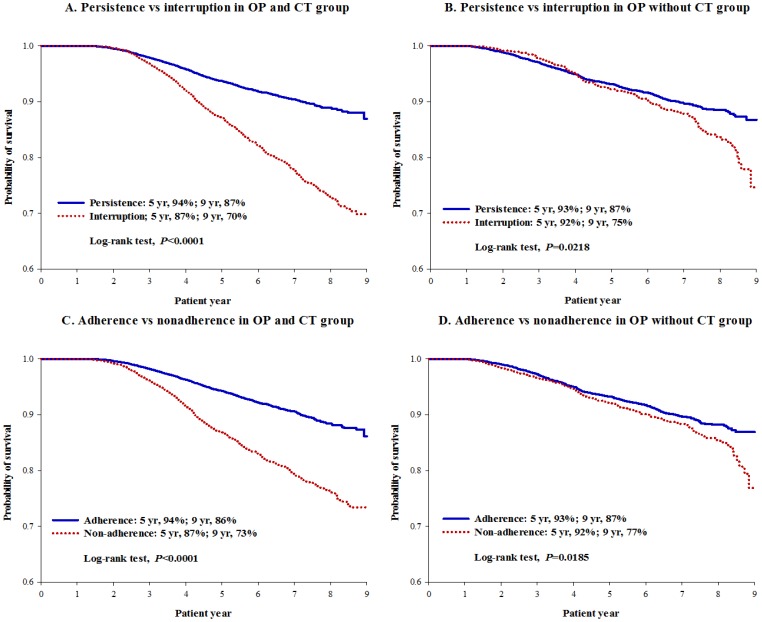
Kaplan-Meier Survival Curves for comparing persistence against interruption and adherence against non-adherence.

For patients receiving OP with adjuvant CT, the HT persistence group had significantly higher 5-year survival rates when compared against the interruption group (94% vs. 87%; Log-rank test, *P*<0.0001) (Figure 1, A). Similar results were also found in patients who did not receive CT (5-year survival rate: 93% vs. 92%; Log-rank test, *P* = 0.0218). However, the differences of survival rates between interruption and persistence groups were smaller in patients without receiving CT than those receiving CT (Figure 1, B). Similar patterns were also noted in comparing HT adherence and non-adherence groups (Figure 1, C and D).

### Mortality Associated with Interruption and Non-adherence

In the multivariable analyses (Table 3), interruption (adjusted HR: 1.32; 95% CI: 1.20, 1.46; *P*<0.0001) and non-adherence (adjusted HR: 1.45; 95% CI: 1.32, 1.59; *P*<0.0001) were significantly associated with increased mortality risk. In addition, higher age ranks, lower income ranks, CCI score≥2, and receiving CT were also significantly associated with an increased mortality risk.

**Table 3:** Univariate and multivariable adjusted hazard ratios of covariates for all-cause mortality.

Characteristic	Univariate hazard ratio	*P* value	Adjusted hazard ratio^a^	*P* value
Persistence	1.00		1.00	
Interruption	2.18 (1.99–2.39)	<0.0001	1.32 (1.20–1.46)	<0.0001
Adherence	1.00		1.00	
Non-adherence	1.98 (1.81–2.16)	<0.0001	1.45 (1.32–1.59)	<0.0001
**Covariates**				
***Age of diagnosis ranks***				
<50 years old	1.00		1.00	
50–64 years old	1.46 (1.32–1.61)	<0.0001	1.29 (1.16–1.44)	<0.0001
65–69 years old	2.19 (1.88–2.55)	<0.0001	1.94 (1.65–2.29)	<0.0001
≥70 years old	3.73 (3.31–4.19)	<0.0001	3.28 (2.84–3.79)	<0.0001
***Initial treatment strategies***				
OP without CT	1.00		1.00	
OP and CT	1.20 (1.09–1.33)	0.0004	1.52 (1.36–1.70)	<0.0001
Radiation therapy				
Without RT	1.00			
With RT	0.98(0.90–1.06)	0.5769	1.16(1.06–1.27)	0.0011
HT utilization pattern				
Tamoxifen only	1.00		1.00	
Tamoxifen to AIs	2.29 (2.07–2.53)	<0.0001	3.48 (3.11–3.89)	<0.0001
AIs only	3.53 (3.12–3.99)	<0.0001	2.98 (2.61–3.41)	<0.0001
AIs to tamoxifen	1.60 (1.04–2.47)	0.0331	1.57 (1.02–2.43)	0.0421
Multiple switches	1.43 (1.19–1.73)	0.0002	2.48 (2.04–3.00)	<0.0001
***CCI score***				
0	1.00		1.00	
1	1.20 (1.07–1.33)	0.0011	1.02 (0.92–1.14)	0.7063
2	2.04 (1.82–2.28)	<0.0001	1.25 (1.11–1.41)	0.0004
***Insurance income ranks*** b				
>20000 NTD^c^ per month	1.00		1.00	
< = 20000 NTD^c^ per month	1.93 (1.73–2.15)	<0.0001	1.48 (1.32–1.65)	<0.0001
No income	1.93 (1.75–2.13)	<0.0001	1.32 (1.19–1.46)	<0.0001

Abbreviations: OP = operation; CT = chemotherapy; RT = radiation therapy; CCI = Charlson Comorbidity Index; NTD = New Taiwan Dollar; HT = hormonal therapy; AIs = aromatase inhibitors.

a Hazard ratios (95% confident interval) were adjusted for all listed variables in the table as well as residential areas of NHI divisions, year of HT initiation, HT prescription duration and time-dependent covariates.

b The income-related insurance payment category set by the Bureau of National Health Insurance in Taiwan.

c 1 NTD = 0.03 USD in 2012.

### Subgroup Analysis

**Figure 2:**
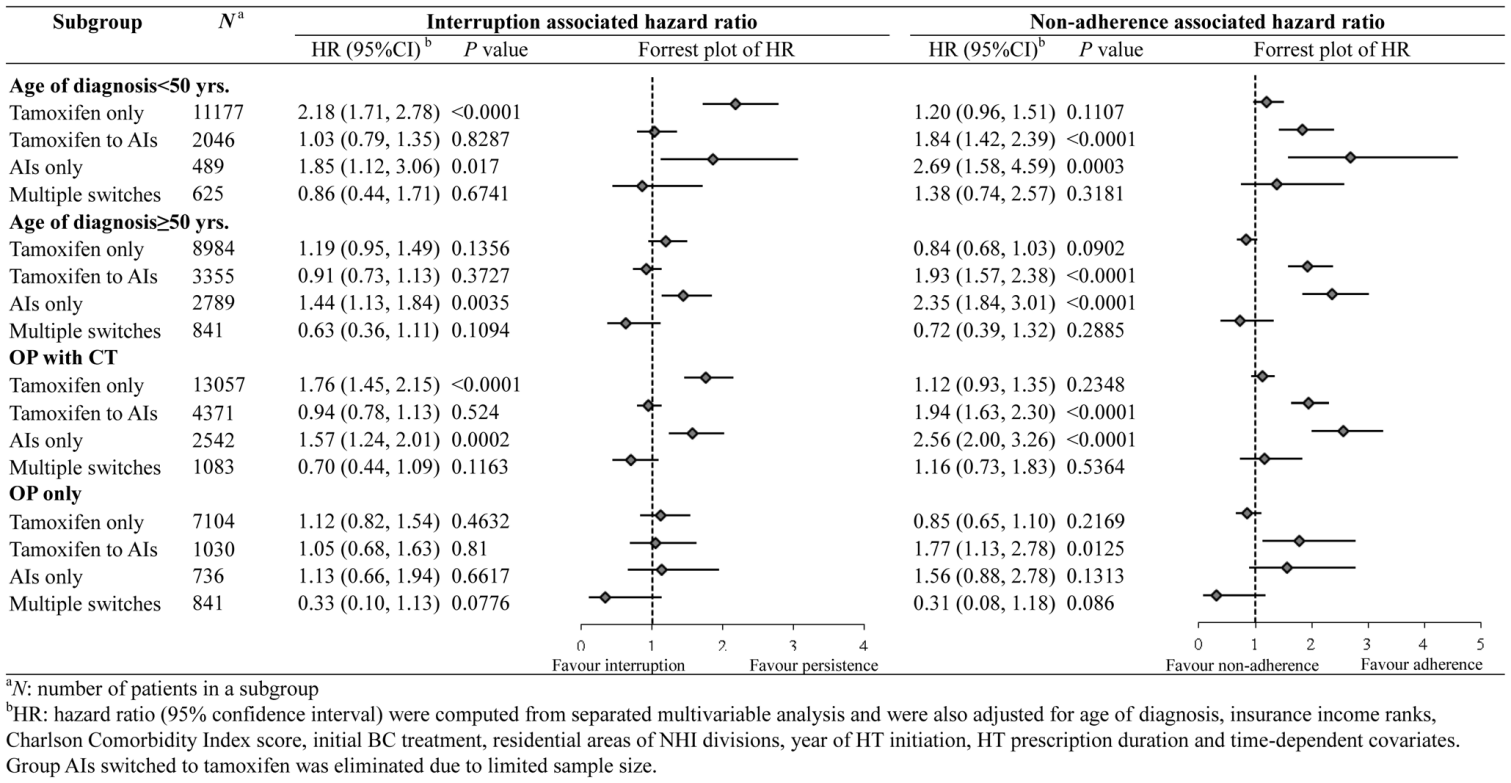
Multivariable adjusted hazard ratios of all-cause mortality for interruption and non-adherence in age and initial treatment strategy subgroups stratified by HT utilization pattern.

The interruption and non-adherence associated mortality risk was further investigated in subgroups according to age, treatment strategy and HT utilization patterns (Figure 2). For interruption, higher risks were observed in the tamoxifen only group of patients diagnosed before 50 years of age (HR: 2.18; 95% CI: 1.71, 2.78). Increased HRs associated with non-adherence were found in patients who received AIs only, regardless of age less than 50 years (HR: 2.69, 95% CI: 1.58, 4.59) or over 50 years (HR: 2.35, 95% CI: 1.84, 3.01) and in the ‘OP with CT’ group (HR: 2.56, 95% CI: 2.00, 3.26).

### Sensitivity Analysis

**Figure 3:**
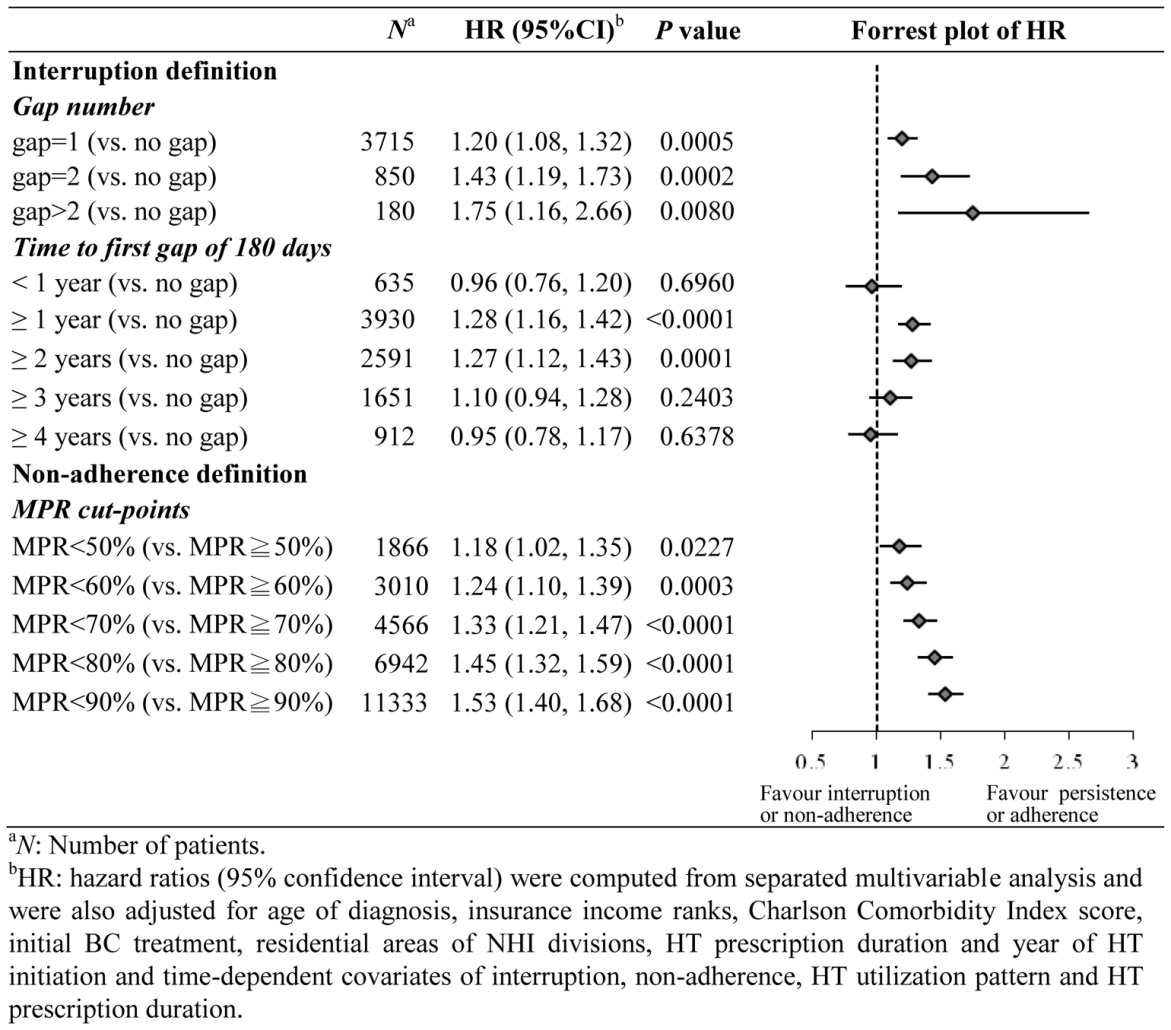
Multivariable adjusted hazard ratios of all-cause mortality by various definitions of interruption and non-adherence.

Multivariable analysis showed that the number of prescription gaps were associated with increased HR in mortality (HR: 1.20 to 1.75). The first interruption occurred after the first (HR: 1.28; 95% CI: 1.16, 1.42) or the second (HR: 1.27; 95% CI: 1.12, 1.43) year of initiating HT was significantly associated with increased mortality risk. In terms of non-adherence, different MPR cut-off levels were associated with increased mortality risk from 50% (HR: 1.18; 95% CI: 1.02, 1.35; *P* = 0.0227) to 90% (HR: 1.53; 95% CI: 1.08, 1.33; *P*<0.0001) (Figure 3).

## Discussion

This population-based study, to our knowledge, is the first to evaluate the impacts of HT interruption and non-adherence on the long-term BC survival using claim-based database in Asia. A study population (*N* = 30,573) who underwent OP and over 12-month adjuvant HT was included. Compared with reports from Western countries [7], [8], this cohort was around 10 years younger (mean diagnosed age: 52 years), yet the 5-year overall survival rates (87% and 94% for patients with and without CT respectively) were similar.

Based on the conventional measures [7], [8], we found 14.9% and 22.7% of interruption and non-adherence rates to HT, and they significantly increased all-cause mortality risk in BC women by 32% (HR: 1.32; 95% CI: 1.20, 1.46) and 45% (95% CI: 1.32, 1.59) respectively. Similar findings were noted by Hershman *et al* (2011) and Makubate *et al* (2013), who reported an increase in mortality rates by 49% and 20% with non-adherence (HR: 1.49, 95% CI: 1.23, 1.81; HR: 1.20, 95% CI: 1.03–1.40) [8], [15].

Interruption and non-adherence measures vary by different definitions and follow-up duration. A systematic review reported a wide range of prevalence of adherence (41∼72%) and non-persistence (31∼73%) to HT measured at the end of 5 years of treatment [16]. By applying various definitions in the sensitivity analysis, we found that interruption-associated mortality increased with increasing interruption frequency, and the non-adherence-associated mortality increased with the higher percentage for MPR cut-off (Figure 3), supporting a dose-response effect of HT on the survival rate.

After adjusting different covariates, this study found elderly age, higher CCI score, lower income, receiving OP and CT (rather than OP alone), and receiving RT were influencing factors to the interruption- and non-adherence-related mortality. Of which, initial treatment strategies and HT utilization patterns may influence on interruption- and non-adherence-related mortality; hence the study cohort was stratified to avoid indication bias.

In line with the National Comprehensive Cancer Network guidelines [17], surgery is recommended as a standard initial treatment for stages I-II BC, and 94% to 97% BC women who received surgery were at stages I-III in Taiwan. Neoadjuvant CT is generally recommended for stage III BC, and adjuvant HT is recommended for hormone receptor positive BC. Similarly, the Taiwan Cancer Registry Report from 2005–2009 also indicated that 89% of patients receiving neo-adjuvant patients are in stage III. Therefore, by defining the cohort as women with newly-diagnosed BC who received OP and HT, most stages I and II BC cases relevant to the study of adjuvant HT would have been included, and those who received both OP and CT could be considered as having tumors with poorer prognosis. Given that adjuvant HT is used in hormone receptor positive BC, the use of additional CT would be a reasonable surrogate indicator of such poor prognosis in this population-based study.

Patients who had CT had greater interruption- and non-adherence-related detrimental effect on survival rates than those who did not have CT (Figure 1). This implies that receiving OP alone could be an indicator for better outcome (indication bias). The notable difference on survival implies that the clinical benefit of HT is more important in patients who received CT (69.4%; this is due to apparently poorer prognosis as explained above).

Previous studies on evaluating the HT discontinuation- or non-adherence-related all-cause mortality were conducted on cohorts with mostly postmenopausal women and with stratification according to HT utilization patterns. [7], [8] About 47% of our BC study cohort was diagnosed at 50 years old or younger, and they had higher proportion of interruption (16.3% vs. 13.8%) and non-adherence (26.3% vs. 19.5%) compared with the older cohort. As the age of 50 years was a surrogate for menopause, we found the impacts of non-adherence and interruptions on mortality HRs were more marked in the premenopausal group (Figure 2).

Non-adherence to AIs may have a greater detrimental effect on survival because AIs have a shorter half-life (24 hours to 50 hours). In contrast, with a longer half-life (5 to 7 days; active metabolite 14 days), non-adherence to tamoxifen, such as delayed or missed doses may not jeopardize the benefit of tamoxifen [18]. However, by stratifying the HT utilization patterns, we found interruption of tamoxifen and non-adherence to AIs were both significantly associated with mortality in the subgroup analysis (Figure 2).

‘Early interruption’ of HT is a concern for BC treatment as the peak recurrence rate has been found to be within the first two years after surgery [19]. A previous study also suggested that patients who ‘consistently’ received 5-year tamoxifen had significantly better event-free survival and overall survival than those who only received 2-years of tamoxifen [20]. Our sensitivity analysis also found the time to the first interruption occurring in the second and third year of HT was significantly associated with increased mortality comparing with the HT persistence group (Figure 3).

We acknowledge several limitations of using a claim-based dataset for medicine and a lack of information on out-of-pocket drug utilization and disease status. Although the out-of-pocket medical utilization is not recorded, as BC is categorized as a ‘catastrophic’ disease and a comprehensive and continuous coverage is provided by the NHI, it is unlikely that BC patients would pay out-of-pocket for the relatively costly, long-term medications (such as AIs). Information on disease status (such as clinical and pathological staging, histological grade and the immunohistochemistry data (ER, PgR and HER2)) were limited, and to reduce the effect of other prognostic factors on our investigation of interruption and non-adherence on mortality, we conducted our analyses within the strata of treatment modality to ensure relatively homogeneous clinical and pathological conditions. In addition, menopausal status was also limited, and hence surrogate indicator, such as cut-off point at median menopausal age of 50 years, was used.

To use MPR as a proxy for adherence, assumptions were made that the dispensing claim data could represent the amount of HT consumed by patients, and methodology commonly used in studies to measure prescription refill adherence in claim-based data [21] have been applied to this study. The definitions of study cohort (patients exposed to HT for at least one year) and the initial treatments (OP and CT recorded within 12 months posterior to the index date) also limited the implications of this study.

Using a predominantly ethnic Chinese women population-based database, we found that interruption and non-adherence to adjuvant HT were independently associated with increase in all-cause mortality. Future research on exploring the reasons for HT interruption and non-adherence is needed in order to develop efficient interventions to ensure completion of recommended course and to gain the best benefit of HT in BC treatment.
